# What's in and what's out in branding? A novel articulation effect for brand names

**DOI:** 10.3389/fpsyg.2015.00585

**Published:** 2015-05-13

**Authors:** Sascha Topolinski, Michael Zürn, Iris K. Schneider

**Affiliations:** ^1^Department of Psychology, University of CologneCologne, Germany; ^2^Department of Psychology, University of WuerzburgWürzburg, Germany; ^3^Department of Psychology, VU University of AmsterdamAmsterdam, Netherlands; ^4^Department of Psychology, University of Southern CaliforniaLos Angeles, CA, USA

**Keywords:** branding, embodiment, phonation, sound symbolism, articulation

## Abstract

The present approach exploits the biomechanical connection between articulation and ingestion-related mouth movements to introduce a novel psychological principle of brand name design. We constructed brand names for diverse products with consonantal stricture spots either from the front to the rear of the mouth, thus inwards (e.g., BODIKA), or from the rear to the front, thus outwards (e.g., KODIBA). These muscle dynamics resemble the oral kinematics during either ingestion (inwards), which feels positive, or expectoration (outwards), which feels negative. In 7 experiments (total *N* = 1261), participants liked products with inward names more than products with outward names (Experiment 1), reported higher purchase intentions (Experiment 2), and higher willingness-to-pay (Experiments 3a–3c, 4, 5), with the price gain amounting to 4–13% of the average estimated product value. These effects occurred across English and German language, under silent reading, for both edible and non-edible products, and even in the presence of a much stronger price determinant, namely fair-trade production (Experiment 5).

## Introduction

The brand name is an important feature of a product substantially shaping consumer's attitudes toward products (e.g., Rao and Monroe, 1989; Fombrun and Shanley, 1990; Keller, 1993; Erdem and Swait, 2004; Elliott and Yannopoulou, 2007; Reimann et al., 2012; Schmitt, 2012). Consumers' knowledge and affective attitudes to brands are determined by a wide range of psychological mechanisms (Keller, 2003), and empirically exploring these mechanisms to inform branding policies has been a lively researched topic in recent years (Keller, 2003; Keller and Lehmann, 2006). For instance, determinants of long-term relations between consumers and brands (Fournier, 1998; Aaker et al., 2004; Aggarwal, 2004), the impact of objectively irrelevant features on attitudes for brands (Meyvis and Janiszewski, 2002; Folkes and Matta, 2004), language-related effects (e.g., Schmitt et al., 1994; Schmitt and Zhang, 1998), or emotions (Swaminathan et al., 2009; Esch et al., 2012; Dunn and Hoegg, 2014) have been examined.

An interesting domain is how features of the brand name itself affect consumers' attitudes. Such effects constitute an articulatory case of *experiential marketing* (Schmitt, 2003), a hidden but direct route to costumers' attitudes via act experiences during the mere reading of a brand name. One example is the fluency of a brand name (Janiszewski and Meyvis, 2001), this is, how easily and efficient a name can be perceived, read, or retrieved (Reber et al., 2004). Basic psychological research has shown that high fluency of words is generally experienced as positive (Topolinski et al., 2009; Topolinski and Reber, 2010a,b; for semantic fluency, see Topolinski and Strack, 2008, 2009a,b,d; Topolinski, 2012a).

One means to increase fluency of a brand name is by simple repetition of that name, such as via advertising (Campbell and Keller, 2003)^1^. Accordingly, it has been shown that repeated exposures of brands do indeed increase positive attitudes and the likelihood of eventual brand choice (e.g., Janiszewski, 1993; Lodish et al., 1995; Baker, 1999; Blüher and Pahl, 2007; Matthes et al., 2007). This effect equals the notion of mere exposure in social psychology (Moreland and Topolinski, 2010; Topolinski, 2013). Most recently, such repetition-induced high fluency due to advertising has been demonstrated to hinge on covert articulation simulations in the mouth, which are trained by repeated exposure and thereby gain in motor fluency: Blocking these subtle mouth exercises, for instance via letting individuals eat popcorn during advertising, prevented advertising effects (Topolinski et al., 2014a; for other interference effects regarding different effectors, see Topolinski and Strack, 2009c, 2010; Topolinski, 2010, 2012b; Sparenberg et al., 2012; Leder et al., 2013). Another means of increasing fluency is pronunciation easiness. For instance, Song and Schwarz (2009) found more positive attitudes toward easy-to-pronounce compared to hard-to-pronounce target words, or Laham et al. (2012) found more positive attitudes toward persons with easy compared to hard-to-pronounce names. Applying this to economic decisions, Alter and Oppenheimer (2006) showed that shares with easy compared to shares with hard-to-pronounce ticker codes showed more positive prize developments, obviously because traders more likely bought them due to verbal fluency.

Another feature of names that affect consumer's responses are phonetic effects, this is, how the name sounds, which has been investigated in research on *phonetic symbolism* (Sapir, 1929; see also Fitch, 1994) or *sound symbolism* (Yorkston and Menon, 2004; Hinton et al., 2006). In these phenomena, the sound of a word conveys certain characteristics of the denoted object or product, such as size, color, or touch. For instance, some vowels sound high (for instance [i] as in SWEET), and other vowels sound low, (for instance [u] as in LOOP). High vowels are associated with little, fast, or light objects, while low vowels are associated with large, steady, or heavy objects (e.g., Klink, 2000; Coulter and Coulter, 2010). As a consequence, Lowrey and Shrum (2007) demonstrated that fictious brand names for hammers (which are heavy) were preferred when they featured low vowels, but brand names for knifes (which are sharp and light) were preferred when they featured high vowels.

Concluding, these earlier contributions on name effects have demonstrated that articulation fluency and word sounds influence consumer choices. Going beyond this, the present paper introduces a novel effect not conceived yet, which is driven by the mere sequence of muscle movements during the articulation of brand names.

### Articulation and ingestion share muscle dynamics

Evolutionary, the oldest function of the oral muscle system is the ingestion of edible and the expectoration of unedible or even harmful substances (Rozin, 1996; Rosenthal, 1999; Duffy, 2007; Hejnol and Martindale, 2008). Ingestion, this is, the intake of foods and liquids, is performed by such activities like licking, sucking, slurping, and swallowing. Functionally, all these mouth movements serve to propel substances from the lips over the tongue into the pharynx and eventually the esophagus (Rosenthal, 1999). Crucially, this propulsion from the front to the rear of the oral cavity necessarily involves a sequence of muscle contractions that start in the front of the mouth –the lips–, over the front of the tongue to the rear of the tongue, similar to the peristalsis of the esophagus (Goyal and Mashimo, 2006; Topolinski and Türk Pereira, 2012). In contrast, the expectoration of unwanted substances, such as during spitting, coughing, or puffing, serves the propulsion of substances from the rear of the mouth toward to front. Biomechanically, these activities require a sequence of muscle tensions starting in the rear of the mouth –the root of the tongue– over the middle and front of the tongue to the lips (Goyal and Mashimo, 2006). Concluding, ingestion requires an inward, and expectoration requires an outward peristaltic wandering of muscle contractions in the mouth.

However, the mouth also serves another, evolutionarily more recent function in humans, namely the faculty of language and speech (Steklis and Harnad, 1976; Rozin, 1999). Speech is realized by the mouth via articulation, and uses the same muscle effectors, namely lips and various spots of the tongue, as ingestion and expectoration (Ladefoged and Maddieson, 1996; Inoue et al., 2007). Crucially, across all languages, the manner of articulation is to modulate or even partly obstruct the airflow from the lungs outside the mouth; and this is done via various muscle contractions (Titze, 2008; Crystal, 2010). For instance, the phoneme [k] as in the English word *C*ONSUMER is produced by pressing the back of the tongue at the soft palate, or the phoneme [ð] as in the English word *TH*ING is produced by pressing the front blade of the tongue against the upper teeth. While the articulation of vowels involve larger muscle parts and even facial muscles, the articulation of consonants requires very specific muscle tensions on well-defined spots. Due to the mouth's anatomy, these places of these *consonantal stricture spots* (e.g., Ladefoged and Maddieson, 1996) are dispersed over the mouth on the sagittal plane, that is, from the front (e.g., the consonants B and P) to the rear (e.g., the consonant K).

Given this, it is possible to construe words that feature consonant sequences that wander either from the front to the rear (inward) or from the rear to the front (outward) of the mouth. Take, for instance, the three consonants K, D, and P. Arranged in the word KADAP, first the rear back of the tongue is pressed against the soft palate to generate K, then the tip of the tongue is pressed against the soft palate to generate D, and then the lips are pressed together to generate P. These muscle tensions thus wander from the rear to the front of the mouth, this is, outward. Reversely, arranged in the word PADAK, first the lips are pressed together, then the tip of the tongue touches the soft palate, and then the rear back of the tongue touches the soft palate. These muscle tensions wander from the front to the rear, of the mouth, that is, inward.

Combining such articulatory patterns with the muscle patterns of ingestion and expectoration, it is obvious that inward consonantal wanderings (PADAK) resemble the muscular dynamics during ingestion, and outward consonantal wanderings (KADAP) resemble the muscular dynamics during expectoration (see for anatomical details, Goyal and Mashimo, 2006). Since ingestion is positively associated, and expectoration is negatively associated (e.g., Rozin, 1996; Rosenthal, 1999), inward consonantal wanderings may feel positive and outward wanderings may feel negative.

### Consonantal wanderings affect attitudes for words

Reading words that systematically feature inward or outward wanderings of consonantal stricture spots might elicit motor patterns similar to ingestion and expectoration, respectively, and might thereby trigger affective responses that ecologically associated with these oral functions, namely brief positive and negative affective states (see, for the notion of brief affective states in general, e.g., Topolinski, 2011, 2014a; Topolinski and Deutsch, 2012, 2013; Topolinski and Strack, 2015). In a recent lines of psychological basic research, this was tested for nonsense words outside a consumer context. Topolinski et al. (2014b) presented inward (e.g., MENIKA) and outward (e.g., KENIMA) words to participants and labeled these as nonsense words that were to be rated for positivity for future studies. Across several experiments in that paper, it was found that participants preferred inward over outward words. This was true if the words were labeled as simple nonsense words without any meaning, but also as person names. Furthermore, the effect occurred even for negatively associated target words: When rating their preference for names of villains in a strategic computer game, participants reported higher liking ratings for inward over outward names (Topolinski et al., 2014b, Experiment 8). However, for attitude objects with disgust associations, such as toxic chemicals, the effect vanishes (Topolinski et al. under revision).

These effects occurred even when participants only silently read those words (Topolinski et al., 2014b). This latter aspect is well in line with earlier evidence that even silent reading triggers covert articulation simulations that engage the mouth (for word repetition, see Topolinski and Strack, 2009c, 2010; Topolinski, 2012b). Also, the effects of phonetic symbolism outlined earlier in the present introduction have been found even under silent reading (Klink, 2000; Coulter and Coulter, 2010). Demonstrating that a covert articulation simulations are the driving mechanism, these effects disappeared under oral motor interference (Topolinski and Bakhtiari, under revision).

### Overview of the experiments

Given the current theorizing and the recent evidence on nonsense words and person names, we predicted that oral inward and outward kinematics in brand names would also influence central measures of consumer attitudes. To test this, we used three central dependent measures of consumer attitudes, namely liking, purchase intentions, and willingness-to-pay. These measures represent different aspects of consumer preferences and therefore allow for a reliable estimation of the articulation effect on actual purchase decisions (Ajzen and Fishbein, 1980). As such, liking captures the hedonic components of preferences (Holbrook and Hirschman, 1982) which are often considered the basis for economic utility and behavior (Bentham, 1789; Becker, 1976; Kahneman et al., 1997). Furthermore, the translation of preferences into actual purchase intentions spreads out onto the behavioral level and thus plays a crucial role for the commercial success of a brand (Ajzen, 1991). In contrast, willingness-to-pay refers to the mapping of a product's utility into the monetary dimension (Hicks, 1946) which allows marketers to gauge the acceptable price range for a product. In sum, this array of dependent measures covers a broad range of consumer reactions toward brands and therefore allows an ecologically valid investigation of the impact of consonantal structure of brand names.

We chose to manipulate consonantal wandering in a within-subjects fashion, presenting many different inward and outward stimuli (as is costume in basic research), since such heuristic judgmental effects work best when participants receive both kinds of stimuli (e.g., for fluency and truth, see Hansen et al., 2008). One might argue that such a set-up reduces the possible managerial implications of the present effect, since brands should benefit from it in a single exposure (which would imply a one-item test). However, judgments about a given target are never rendered in isolation but in psychological relativity to other stimuli (Mussweiler, 2003), so are consumer judgments: Our set-up thus instantiates the everyday situation where we find many different providers and brands for a certain product category we want to purchase, and we chose from this array of options.

## Experiment 1

In this first experiment, the impact of consonantal inward and outward wanderings in brand names on consumer attitudes was tested. In a market survey, possible brands for several products were presented to participants, who reported their brand attitude by indicating how much they liked each potential brand name (Petty et al., 1983; Janiszewski, 1993; Oliver, 1999). To cover a range of products to demonstrate the generalizability of the present effect, we arbitrarily chose four rather heterogeneous products. Since the recent trend of exploding numbers of digital and communication products force providers to come up with ever new exotic names for their products that are artificially created (e.g., SKYPE, NAPSTER, AMPYA, or SHAZAM), we chose the product categories of mobile application software and antivirus software as products for which consumers are used to artificially created nonsense names as brands. Because generic drugs also usually feature a bulk of artificially created names, we chose pain killer drugs as a third category. To test whether also negatively associated, or even disgust-related, products would also be affected by name features, we chose pest control as a fourth product category, since chemicals also commonly feature names that are nonsense to non-experts.

### Methods

#### Data treatment

For all studies in the present paper, we report all measures and conditions that were run in the single experiments. We report and justify exclusion of data.

We calculated required sample sizes a-priori using G^*^Power (Faul et al., 2007). We used the effect size obtained in the meta-analysis in Topolinski et al. (2014b) for the basic inward vs. outward effect on liking, *d_z_* = 0.40. To replicate this effect two-sided with a power of 0.80, required sample size is *N* = 52. Because we did not know about the effect on the current measures of consumer attitudes, we arbitrarily set the sample sizes higher. Thus, most of the present experiments are over-powered. For all experiments, analyses were run only after the full final sample size had been collected.

#### Participants

A total of *N* = 402 (182 female, 219 male, 1 not identified, *M_age_* = 33, *SD_age_* = 11) participants from the US were recruited via Amazon mTurk and received $0.5 for the 5 min online experiment. Thirty participants (7%) were discarded from the later analyses because they reported a different language than English or failed to remember the product at the end of the session (see the following Method section), resulting in an ultimate sample of *N* = 372.

#### Materials and procedure

We used 125 inward and 125 outward words from the stimulus pool used by Topolinski et al. (2014b; Experiment 6; stimulus pool D) that had been created to induce inward and outward wanderings of consonantal stricture spots in articulation. The pool is available online as supplementary online material for the original article (http://dx.doi.org/10.1037/a0036477.supp). This pool had been created the following way. Consonants were sampled from three consonant groups that features well-defined articulation spots, namely in the front (labial and labio-dental consonants: B, F, M, P), the middle (alveolar consonants: D, L, N, S, T), and rear (velar-uvular: K) of the mouth. From these three groups, all possible inward combinations of consonants (that is, front-middle-rear) were generated (e.g., BDK). Into these strings, random vowels were inserted after each consonant (e.g., BODEKA). Matching outward words were generated by simply reversing the consonantal sequence but leaving the vowel sequence intact, such as BODEKA to KODEBA. Resulting words with meaningful syllables (e.g., USA) were discarded. Example words are APODOKE, BODEK, IBUSEK, and UMALAKO as inward words, or AKENUPE, IKUTEM, KONOM, and UKANAMO as outward words.

In the survey, participants first read a brief instruction (full materials, see Supplementary Material) where the respective product category (antivirus, app, pest control, or painkiller) was mentioned and then indicated how much they liked each potential brand name on a scale ranging from 0 (not at all) to 10 (very much so). Each participant rated a sequence of 50 names. Twenty-five names were randomly drawn from the inward pool (125 items) and 25 names were randomly drawn from the outward pool (125 items). The order of inward and outward names was completely random, randomized anew for each participant.

After completing the survey, participants reported age and gender; and were asked for the product they had been rated before. If the participant did not type in the correct product category, his or her data was discarded. This way, we assured that participants had the product in mind during the whole session.

### Results and discussion

The mean liking ratings for all products and consonantal conditions are shown in Table 1. A 2 (Consonantal Stricture Direction: inward, outward; within) X 4 (Product: antivirus software, pain killer drug, pest control, mobile application; between) analysis of variance (ANOVA) with the first factor as repeated measures factor on the averaged liking ratings found a main effect for Consonantal Direction, *F*_(1, 368)_ = 26.48, *p* < 0.001, η^2^*_p_* = 0.07, and a main effect for Product, *F*_(3, 368)_ = 2.67, *p* = 0.047, η^2^*_p_* = 0.02, but no interaction (*p* = 0.58). The main effect of Consonantal Direction was constituted by the fact that across all products, inward brand-names (*M* = 3.70, *SE* = 0.08) were preferred over outward brand-names (*M* = 3.55, *SE* = 0.08), *t*_(371)_ = 5.19, *p* < 0.001, *d_z_* = 0.27, 95% CI [0.10, 0.21]. The conceptually irrelevant main effect of Product Type was constituted by the fact that pain killers and mobile apps were preferred over antivirus software and pest control, probably because the former are more frequently purchased and more common than the latter.

**Table 1:** **Summary of experiments and results**.

**Experiment DV Scale**	**Sample**	**Stimulus pool and number of stimuli presented**	**Product**	**Results (Means and) standard errors**	
				**Inward**	**Outward**
**EXPERIMENT 1**					
Liking (0–10)	*N* = 402 English (online)	English pool	Antivirus (*n* = 102)	3.42 (0.15)	3.26 (0.14)
	182 female, 219 male, 1 not identified	25 inward words	Painkiller (*n* = 96)	3.88 (0.15)	3.69 (0.15)
	*M_age_* = 33, *SD_age_* = 11	25 outward words	Pest control (*n* = 83)	3.62 (0.16)	3.45 (0.15)
			Mobile app (*n* = 91)	3.90 (0.17)	3.82 (0.16)
**EXPERIMENT 2**					
Purchase likelihood (1–9)	*N* = 202 English online	English pool	Antivirus (*n* = 44)	3.27 (0.19)	3.26 (0.14)
	74 female, 127 male, 1 not identified	25 inward words	Painkiller (*n* = 43)	3.82 (0.18)	3.69 (0.15)
	*M_age_* = 32, *SD_age_* = 11	25 outward words	Pest control (*n* = 48)	3.34 (0.17)	3.45 (0.15)
			Mobile app (*n* = 91)	3.13 (0.22)	3.82 (0.16)
**EXPERIMENTS 3A–3C WILLINGNESS-TO-PAY (0–500 CENTS)**					
Experiment 3a	*N* = 127 German onsite	German pool	Chocolate bar	92 cents (4)	78 cents (4)
	90 female, 34 male, 3 missing reports	10 inward words			
	*M_age_* = 27, *SD_age_* = 9	10 outward words			
Experiment 3b	*N* = 102 German onsite	German pool	Chocolate bar	77 cents (5)	67 cent (5)
	67 female, 35 male, 3 missing reports	10 inward words			
	*M_age_* = 23, *SD_age_* = 6	10 outward words			
Experiment 3c	*N* = 86 German onsite	German pool	Chocolate bar	102 cents (8)	95 cents (8)
	44 female, 42 male	20 inward words			
	*M_age_* = 24, *SD_age_* = 4	20 baseline words			
		20 outward words			
**EXPERIMENT 4**					
Willingness-to-pay (0–500 cents)	*N* = 53 English online	English pool	Painkiller	107 cents (13)	99 cents (13)
	20 female, 33 male	25 inward words			
	*M_age_* = 29, *SD_age_* = 8	25 outward words			
**EXPERIMENT 5**					
Willingness-to-pay (0–500 cents)	*N* = 289 German onsite	German pool	Chocolate bar fair-trade	151 cents (5)	146 cents (4)
	223 female, 48 male, 18 missing reports	10 inward words			
	*M_age_* = 23, *SD_age_* = 6, 4 missing reports	10 outward words	Chocolate not fair-trade	89 cents (3)	84 cents (3)

These findings show that consonantal structures in brand names can indeed shape product attitudes, which was even true for negatively associated products such as pest control.

## Experiment 2

Experiment 2 was designed for an initial investigation of the behavioral aspects of brand name articulation. Therefore, we replicated Experiment 1 with purchase intentions as dependent measure (Oliver, 1980; Chen et al., 1998; Newman et al., 2011).

### Methods

#### Participants

A total of *N* = 202 (74 female, 127 male, 1 not identified, *M_age_* = 32, *SD_age_* = 11) participants from the US were recruited via Amazon mTurk and received $0.5 for the 5 min online experiment. Twenty-seven participants (13%) reported a language different than English or were unable at the end of the session to recall the product type correctly they had been asked to rate. Their data were discarded resulting in an ultimate sample of *N* = 175.

#### Materials and procedure

Experiment 1 was replicated with the only modification that participants were asked to report how likely they would purchase the product on a scale from 1 (not likely) to 9 (very likely) (full materials, see Supplementary Material).

### Results and discussion

The mean purchase likelihoods are presented in Table 1. A 2 (Consonantal Stricture Direction: inward, outward; within) × 4 (Product: antivirus software, pain killer drug, pest control, mobile application; between) ANOVA on these averaged purchase likelihoods found again a main effect for consonantal direction, *F*_(1, 171)_ = 4.37, *p* = 0.038, η^2^*_p_* = 0.03, and a marginal main effect for product, *F*_(3, 171)_ = 2.64, *p* = 0.051, η^2^*_p_* = 0.04, but again no interaction (*p* = 0.98). Collapsed over products, participants reported a higher likelihood of purchasing a product with an inward brand-name (*M* = 3.39, *SE* = 0.09) than an outward brand-name (*M* = 3.31, *SE* = 0.09), *t*_(174)_ = 2.09, *p* = 0.038, *d_z_* = 0.16, 95% CI [0.00, 0.17]. The conceptually irrelevant main effect of product type was constituted by the fact that pain killers elicited higher purchase likelihood than the other products.

## Experiments 3a, 3b, and 3c

Experiments 1 and 2 have shown the existence of an articulation effect in the context of brand names by implementing psychological measures widely used in basic consumer research. However, referring to “hard numbers” can be vital for a successful internal communication of new marketing strategies. Therefore, in three further experiments we measured consumers' willingness-to-pay for a certain brand as a function of brand name articulation. Willingness-to-pay directly measures monetary product evaluation (Adaval and Wyer, 2011; Bornemann and Homburg, 2011; Palmeira and Srivastava, 2013) and provides useful information on the actual economic relevance of marketing decisions, this is, how much may the brand name contribute to the profitability of a product. To generalize to another product category, we chose a chocolate bar as product, also because this edible product is highly associated to the oral domain and related oral consumption responses. In Experiment 3a we used a paper-pencil questionnaire, in 3b a computer-directed survey, and in Experiment 3c we included a baseline with names that showed mixed inward and outward transitions across their consonants. This baseline informs us how systematic inward and outward transitions influence willingness to pay relative to common language, which usually shows no systematic but random wanderings.

### Methods

#### Participants

In Experiment 3a, *N* = 127 (90 female, 34 male, 3 missing reports, *M_age_* = 27, *SD_age_* = 9), Experiment 3b, *N* = 102 (67 female, 35 male, 3 missing reports, *M_age_* = 23, *SD_age_* = 6), and in Experiment 3c, *N* = 86 (44 female, 42 male, *M_age_* = 24, *SD_age_* = 4) German speaking volunteers from various professional backgrounds from the city area of Würzburg (Experiments 3a, 3c) and non-psychology undergraduate from the University of Cologne (Experiment 3b) participated as part of larger experimental batteries involving other unrelated tasks (ratings jokes, Topolinski, 2014b; rating geometric figures, Topolinski et al., 2015) receiving €8 as financial compensation for their participation.

#### Materials

In Experiments 3a and 3b, due to practical and time factors (paper pencil questionnaire, brief task required) we used the small stimulus pool designed for German articulation from Topolinski et al. (2014b, Experiments 1–2). These words feature also the consonants R and G as clearly velar (i.e., rear) consonants in German articulation. In English articulation, the letter G has several possible articulation spots depending on surrounding vowels and consonants (e.g., GUM in the rear vs. GINGER in the front), but in German articulation this letter is always articulated velar, that is, in the rear (phoneme [g]). The letter R is generally pronounced as an alveolar phoneme in English ([ɹ]) being generated with the front of the tongue. However, in German articulation it is usually a uvular (i.e., very rear) phoneme ([R] or [[ʁ]], cf., French R) being generated with the rear dorsum of the tongue tapping against the very rear soft palate. Thus, the articulation of the German R is even more in the rear than the articulations of K and G, which allowed an ever stronger manipulation of inwards and outwards dynamics by adding R as most extreme rear category. The respective stimuli were 10 inward-words (BALUGOR, BATIKERO, BULEKA, MADOGU, MENIKA, MESUKIRO, MUSAGI, PANOKARE, PATUGI, PODAKERI) and 10 outward-words (RAGULOB, RAKITEBO, KULEBA, GADOMU, KENIMA, REKUSIMO, GUSAMI, RAKONAPE, GATUPI, ROKADEPI). In Experiment 3c, the large stimulus pool of 120 inward, 120 outward, and 120 baseline words developed in Topolinski et al. (2014b, Experiment 5) was used. The baseline pool of words was constructed by flipping two random consonants in each of the original inward words. For instance, from the inward word FOLOK a baseline word was derived by simply flipping L and F, resulting in LOFOK (first outward-transition from L to F, and then inward transition from F to K).

#### Procedure

In Experiment 3a, after having completed several unrelated computer-directed experimental tasks participants were given a one-page paper-pencil questionnaire with the stimuli printed in one random order similar for all participants and were asked to fill it out (see Supplementary Material). In Experiment 3b, after having completed several unrelated computer-tasks participants received the stimuli in a computer-directed survey in random order re-randomized anew for each participants. In Experiment 3c, after having completed another unrelated task, participants received the stimuli in a computer-directed survey (with the same instructions). For each participant anew, 20 inward, 20 baseline, and 20 outward words were randomly sampled from the larger stimulus pools and were presented in random order. The instruction was identical in all experiments (see Supplementary Material). Participants were informed that this would be a market survey on possible names for novel brands for chocolate bars (100 g weight). They were asked to report how much they would be willing to pay in a range from 0 to 500 cents and were asked to report their estimate in cents (e.g., to report 155 if they were willing to pay €1.55).

### Results and discussion

In Experiment 3a, the mean WTP ratings were 92 cents (*SE* = 4) for inward brands and 78 cents (*SE* = 4) for outward brands. Thus, participants were willing to pay 14 cents more for inward than for outward brands, *t*_(126)_ = 6.57, *p* < 0.001, *d_z_* = 0.58, 95% CI [10.10, 18.81]. In Experiment 3b, the mean WTP ratings were 77 cents (*SE* = 5) for inward brands and 67 cents (*SD* = 5) for outward brands. Here, participants were willing to pay 10 cents more for inward than for outward brands, *t*_(101)_ = 4.19, *p* < 0.001, *d_z_* = 0.41, 95% CI [5.08, 14.22]. In Experiment 3c, the mean WTP ratings were 102 cents (*SE* = 8) for inward brands, 101 cents (*SE* = 8) for control brands, and 95 cents (*SD* = 8) for outward brands. Participants were willing to pay 7 cents more for inward than for outward brands, *t*_(85)_ = 3.00, *p* = 0.004, *d_z_* = 0.32, 95% CI [2.41, 11.90], and 6 cents more for control than for outward brands, *t*_(85)_ = 2.81, *p* = 0.006, *d_z_* = 0.30, 95% CI [1.89, 11.03], while inward and control brands did not differ from each other (*t* < 0).

To have a more accurate estimate of the total effect size, combining all three studies, participants were willing to pay 11 cents more for inward compared to outward brands, *t*_(314)_ = 8.16, *p* < 0.001, *d* = 0.46, 95% CI [8.28, 13.54]. This effect size even comes close to the originally found effect size of *d_z_* = 0.40 for simple liking ratings in Topolinski et al. (2014b).

This finding illustrates immediate practical consequences of exploiting consonantal articulation dynamics in branding. To illustrate the possible profit gain, consider the following. Averaged over all three samples and across inward and outward brands, participants were generally willing to pay 84 cents (*SD* = 56) for a chocolate bar. The difference of 11 cents due to inward vs. outward naming thus amounts to 13% of this average monetary evaluation. It is probably hard to find any other marketing variable that is able to produce a comparable gain in revenue at a comparably low cost. The next experiment generalized this effect to an English speaking sample and a larger stimulus pool.

## Experiment 4

The current theorizing holds that inward and outward consonantal wanderings during articulation gain their positivity or negativity from their motor similarity to consumption behaviors, that is, eating, drinking, swallowing, or spitting. However, this attitudinal effect need not be limited to edible products (see Experiment 1). Thus, we generalized the impact of inward and outward branding on willingness-to-pay to the exemplary product category of pain killers.

### Methods

#### Participants

A total of *N* = 53 (20 female, 33 male, *M_age_* = 29, *SD_age_* = 8) participants from the US were recruited via Amazon mTurk and received $0.5 for the 5 min online experiment. Seven participants (13%) reported a different language than English or could not recollect the product type they were asked to rate at the end of the session. Their data were discarded, which yielded a final sample of *N* = 46.

#### Materials and procedure

The procedure was identical to Experiment 2 but participants indicated their willingness-to-pay for a package of pain killers using a slider ranging from 0 to 500 US-Cent (materials see Supplementary Material).

### Results and discussion

Participants reported higher WTP for pain killers with brand names being inward words (107 cents, *SE* = 13) than outward words (99 cents, *SE* = 13), *t*_(45)_ = 2.54, *p* = 0.015, *d_z_* = 0.37, 95% CI [1.51, 13.00]. The nominal effect amounted to 7 cents, which are 7% of the average reported WTP prize for the pain killer being 103 cents (*SE* = 13).

Although the current effect was smaller than in Experiments 3a and 3b (which might be due to the different product, language, or stimulus pool that did not feature the letter R as an extremely rear consonant), it was still reliable and shows that only the brand name may increase consumers' willingness-to-pay by 7%.

## Experiment 5

In the experiments thus far, the brand name was the only information conveyed about the respective products. Although the brand name often is the only cue available, the question is whether the subtle heuristic or peripheral cue of articulation dynamics would still have an effect when other, more systematic or central cues about the product are available (Petty et al., 1983; Strack et al., 2006). We chose fair-trade production (Vantomme et al., 2006) for chocolate as a very strong systematic price cue. Our reasoning was *not* that articulation direction would outperform that substantial cue—of course, fair-trade production would much more substantially impact willingness-to-pay. Rather, the aim was to show that articulation direction would still persist to exert an influence even in the presence of that much more powerful price cue. We could have easily chosen another central cue of minor price impact, such as land of origin of the cacao, but the aim was to let the articulation effect compete with a very strong price determinant. This competition with another strong judgmental influence also goes beyond the earlier demonstrations by Topolinski et al. (2014b).

### Methods

#### Participants

A total of *N* = 289 (223 female, 48 male, 18 missing reports, *M_age_* = 23, *SD_age_* = 6, 4 missing reports) students from freshmen courses took part.

#### Materials and procedure

The brief stimulus pool from Experiments 3a and 3b was used again in a similar paper-pencil questionnaire (instructions see Supplementary Material). Participants were again simply asked how much they would be willing to pay for each sort of chocolate bar. Orthogonally to inward and outward direction of the brand name, to one random half of the brands in a random sequence the information “fair-trade” was added, and to the other half the information “no fair-trade” was added (inward/outward and fair-trade/no-fair-trade assignments counter-balanced across participants). These questionnaires were handed to attendees of various courses and lectures to be filled out in silence.

### Results and discussion

A 2 (Consonantal Stricture Direction: inward, outward; within) X 2 (Fair-trade: fair-trade product, no fair-trade product) ANOVA on the averaged WTP reports detected a very strong main effect of fair-trade, *F*_(1, 288)_ = 304.35, *p* < 0.001, η^2^*_p_* = 0.51, and a reliable effect of consonantal direction, *F*_(1, 288)_ = 15.27, *p* < 0.001, η^2^*_p_* = 0.05, and no interaction (*F* < 1). Generally, participants were willing to pay 62 cents more for a fair-trade (148 cents, *SE* = 4) than for a no-fair-trade chocolate, (87 cents, *SE* = 2). Despite this strong effect, articulation direction impacted WTP for both types of products. Participants reported higher WTP for inward vs. outward brands both for fair-trade (151 cents, *SE* = 5 vs. 146 cents, *SE* = 4) and for no-fair-trade products (89 cents, *SE* = 3 vs. 84 cents, *SE* = 3), both *t*s > 2.4, *p*s < 0.017, *d_z_*s > 0.14. This price gain due to articulation dynamics amounted to 6 cents (4% of the average price) for fair-trade and 4 cents (5% of the average price) for no-fair-trade products.

Thus, despite the presence of a competitive and substantial price determinant, fair-trade production, articulation direction still exerted an influence on willingness-to-pay, with an effect size even comparable to Experiment 4, where no additional product information was given.

## General discussion

The present approach introduces a novel articulation effect for brand names exploiting consonantal sequences that wander inwards and outwards in the mouth during merely reading a brand name. For inward compared to outward product and brand names, participants liked them more, reported higher likelihood to purchase, and higher willingness-to-pay, amounting to up to 13% of the product's estimated value. These effects were robust for both English and German speaking individuals and different set-ups, namely laboratory experiment, online survey, and paper-pencil questionnaires. Also, articulation direction still influenced willingness-to-pay in the presence of another, much stronger price predictor, namely fair-trade production. These findings advise the costless marketing strategy of avoiding outward names (see the baseline comparison in Experiment 3c) yielding an essentially costless monetary gain that is probably unmatched by any other costless marketing strategy.

This strategy is all the more relevant given the recent explosion of new multi-media and lifestyle products, such as mobile application software or e-commerce companies, that do not feature anymore the conservative type of brand names often being the company founder's name (such as Kellogg's ^©^), but ever newly invented fancy and entertaining artificial words, such as SKYPE, NAPSTER, AMPYA, or SHAZAM. Particularly in such markets featuring steady innovation and short product life cycles generating appealing new names is of utter importance to remain competitive, and the present articulation effect seems to be an effective way to increase consumer's brand attitudes. Furthermore, some products (such as raw materials) are often purchased via lists featuring some technical specifications, prices, and names. Here, if two products are basically identical in costs and technical specs having selected the right consonantal structure may very much be the tip of the scale.

Experiment 3c compared this in-out effect on willingness-to-pay to baseline control words that did not feature any systematic wandering of consonantal articulation spots (cf., Topolinski et al., 2014b; Experiment 5). This is particularly interesting because common language usually does not feature systematic wanderings. The finding was that inward brands evoked a similar level of willingness-to-pay as control words, and only outward words fall below both inward and control words. This suggests that marketing strategists might not necessarily seek profit gain from designing inward rather than unsystematic names, but can definitely avoid loss of profit by accidentally choosing an outward brand name, such is in the well-known cases of KAZAM^©^, GANT^©^, or KRAFT^©^.

For global marketing strategies, of course, branding should consider differing phonation of letters in different languages (see, for intercultural effects on branding, e.g., Salciuviene et al., 2010). As a result of differences in speech generation, the same consonant combination may produce a strong inward wandering in some languages, but not in others (see the case of G and R in the English vs. German phonation, Experiments 3a–3c). Brand name design should take this into account.

Furthermore, particularly brand name designing strategies should take into account possible matching effects with the sort of the denoted product. Whereas we found reliable in-over-out preference for all the products used in the present experiments, this is, also for inedible (e.g., software) or even mildly negative products (e.g., pest control), Topolinski et al. (under revision) found an interaction with extreme valences. For toxic chemicals, which presumably activate an immediate disgust response, the in-out effect was attenuated or even reversed. This implies that the presently featured brand name design strategy is only effective for neutral or positive objects. Future research should investigate matching effects with object meaning more closely.

Concluding, the present approach exploits the biomechanical connection between articulation and ingestion to introduce a novel psychological principle for brand name design. Brands for which the consonantal articulation spots wander inwards in the mouth compared to outwards are preferred, elicit higher purchase intentions, and even trigger higher willingness-to-pay with a substantial possible monetary gain.

### Conflict of interest statement

The authors declare that the research was conducted in the absence of any commercial or financial relationships that could be construed as a potential conflict of interest.

## Footnotes

^1^Another elegant means of fluency by repetition might be to repeat single syllables within a brand name (e.g., CO-ca CO-la). We thank one reviewer for this intriguing idea. However, the authors are not aware of any such empirical demonstration. In the present argumentation, we refer to repetition as repetition of the whole brand name.
