# Supplementary material for: What's in and what's out in branding? A novel articulation effect for brand names
Source: Front Psychol. 2015 May 13;6:585. doi: 10.3389/fpsyg.2015.00585 (PMC4429570; doi:10.3389/fpsyg.2015.00585)
Supplement: Supplementary file 1 [file DataSheet1.DOCX]

**Appendix**

Instructions in Experiment 1

„Dear participant,

thank you for helping us in this survey. We are testing possible brand names. In the following you will be presented with 50 candidates of future names of ___. Just read each name and then indicate spontaneously how much you like it. Type in a number between 0 (“I do not like it at all”) to 10 (“I like it very much”).

Just react spontaneously using your guts but keep sure that you really read the name and open yourself to it.”

For ___, depending on the product condition, the words “antivirus software”, “cellphone-applications”, “pest control”, or “painkillers”, were inserted.

In each trial, besides the target word the question appeared: “How much do you like this name as a brandname of ___?”. Again, depending on the product condition, the words “antivirus software”, “cellphone-applications”, “pest control”, or “painkillers”, were inserted for ___.

The scale anchors were 0 “not at all” and 10 “very much so”.

Instructions in Experiment 2

„Dear participant,

thank you for helping us in this survey. We are testing possible brand names. In the following you will be presented with 50 candidates of future names of ___. Just read each name and then indicate spontaneously how likely it is for you to purchase a ___ with the respective name. Type in a number between 1 (not likely) to 9 (very likely).

Just react spontaneously using your guts but keep sure that you really read the name and open yourself to it.”

For ___, depending on the product condition, the words “antivirus software”, “cellphone-applications”, “pest control”, or “painkillers”, were inserted.

In each trial, besides the target word the question appeared: “How likely would you buy a ___ with this name?”. Again, depending on the product condition, the words “antivirus software”, “cellphone-applications”, “pest control”, or “painkillers”, were inserted for ___.

The scale anchors were 9 “not likely” and 10 “very likely”.

Instructions in Experiments 3a, 3b, and 3c

“Liebe Teilnehmer,

nun kommt noch eine kurze Verbraucherumfrage. Wir testen hier mögliche Markennamen für neue Schokoladensorten vor. Lesen Sie jeden Namen durch und geben Sie dann ganz spontan an, wieviel Sie für diese Schokolade jeweils bezahlen würden. Es handelt sich um eine normale Tafel Schokolade mit 100 Gramm Gewicht.

Bitte geben Sie einen Wert in Cents ein von 0 (wenn Sie gar nichts dafür bezahlen würden) bis zu 500 (also 5 Euro).

Bitte geben Sie den Preis in Cents an, also beispielsweise 155 für 1,55 €. Schreiben Sie den Wert einfach in die freie Zeile neben dem Namen!“

[“Dear Participant,

this is a brief consumer survey. We test possible brand names for new sorts of chocolate bars. Please read each name and then report spontaneously how much you would be willing to pay for the given sort. The chocolate bar refers to a normal bar of 100 grams weight.

Please write down the value in cents from 0 (if you would not be willing to pay anything for it) to 500 (this is, 5 Euros).

Please report the price in cents, for, instance, 155 for €1.55. Just write down the value into the blank space provided beside each of the names.”]

Layout of the paper-pencil version in Experiments 3a and 3b

Alter: ____ Geschlecht: m / w

Liebe Teilnehmer,

nun kommt noch eine kurze Verbraucherumfrage. Wir testen hier mögliche Markennamen für neue Schokoladensorten vor. Lesen Sie jeden Namen durch und geben Sie dann ganz spontan an, wieviel Sie für diese Schokolade jeweils bezahlen würden. Es handelt sich um eine normale Tafel Schokolade mit 100 Gramm Gewicht.

Bitte geben Sie einen Wert in Cents ein von 0 (wenn Sie gar nichts dafür bezahlen würden) bis zu 500 (also 5 Euro).

Bitte geben Sie den Preis in Cents an, also beispielsweise 155 für 1,55 €. Schreiben Sie den Wert einfach in die freie Zeile neben dem Namen!

Balugor _______

Rakitebo _______

Batikero _______

Kenima _______

Musagi _______

Rakonape _______

Buleka _______

Mesukiro _______

Panokare _______

Rokadepi _______

Gadomu _______

Patugi _______

Gusami _______

Kuleba _______

Podakeri _______

Ragulob _______

Rekusimo _______

Gatupi _______

Madogu _______

Menika _______

*Vielen Dank für diese Angaben!*

Instructions in Experiment 4

„Dear participant,

thank you for helping us in this survey. We are testing possible brand names for pain killer drugs. In the following you will be presented with several candidates of future names of pain killers. Just read each name and then indicate the maximum amount you would be willing to pay for this product from 0 to 500 US cents (this is, $5).

Just react spontaneously using your guts but keep sure that you really read the name and open yourself to it.”

In each trial, besides the target word the question appeared “What is the maximum amount you would be willing to pay for this product?” along with a continuous slider ranging from 0 to 500 US-Cent.

Paper-pencil questionnaire used in Experiment 5

Version A:

Alter: ____ Geschlecht: m / w

Liebe Teilnehmer,

dies ist eine kleine Verbraucherumfrage für verschiedene neue Namen von Schokoladensorten. Manche von ihnen sind Fairtrade und manche sind nicht Fairtrade. Lesen Sie jeden Namen und die zusätzliche Information und geben Sie dann ganz spontan an, wieviel Sie für diese Schokolade jeweils bezahlen würden. Es handelt sich um eine normale Tafel Schokolade mit 100 Gramm Gewicht.

Bitte geben Sie einen Wert in Cents ein von 0 (wenn Sie gar nichts dafür bezahlen würden) bis zu 500 (also 5 Euro).

Bitte geben Sie den Preis in Cents an, also beispielsweise 155 für 1,55 €. Schreiben Sie den Wert einfach auf die Linie neben dem Namen!

Balugor *no-fairtrade* _______

Rakitebo *fairtrade* _______

Batikero *fairtrade* _______

Kenima *no-fairtrade* ______

Musagi *fairtrade* _______

Rakonape *no-fairtrade* _______

Buleka *no-fairtrade* _______

Mesukiro *fairtrade* ______

Panokare *no-fairtrade* _______

Rokadepi *no-fairtrade* _______

Gadomu *fairtrade* _______

Patugi *no-fairtrade* _______

Gusami *fairtrade* _______

Kuleba *fairtrade* _______

Podakeri *no-fairtrade* _______

Ragulob *fairtrade* _______

Rekusimo *no-fairtrade* _______

Gatupi *no-fairtrade* _______

Madogu *fairtrade* _______

Menika *fairtrade* _______

*Vielen Dank für diese Angaben!*

Version B:

Alter: ____ Geschlecht: m / w

Liebe Teilnehmer,

dies ist eine kleine Verbraucherumfrage für verschiedene neue Namen von Schokoladensorten. Manche von ihnen sind Fairtrade und manche sind nicht Fairtrade. Lesen Sie jeden Namen und die zusätzliche Information und geben Sie dann ganz spontan an, wieviel Sie für diese Schokolade jeweils bezahlen würden. Es handelt sich um eine normale Tafel Schokolade mit 100 Gramm Gewicht.

Bitte geben Sie einen Wert in Cents ein von 0 (wenn Sie gar nichts dafür bezahlen würden) bis zu 500 (also 5 Euro).

Bitte geben Sie den Preis in Cents an, also beispielsweise 155 für 1,55 €. Schreiben Sie den Wert einfach auf die Linie neben dem Namen!

Balugor *fairtrade* _______

Rakitebo *no-fairtrade* _______

Batikero *no-fairtrade* _______

Kenima *fairtrade* _______

Musagi *no-fairtrade* _______

Rakonape *fairtrade* _______

Buleka *fairtrade* _______

Mesukiro *no-fairtrade* ____

Panokare *fairtrade* _______

Rokadepi *fairtrade* _______

Gadomu *no-fairtrade* _______

Patugi *fairtrade* _______

Gusami *no-fairtrade* _______

Kuleba *no-fairtrade* _______

Podakeri *fairtrade* _______

Ragulob *no-fairtrade* _____

Rekusimo *fairtrade* _______

Gatupi *fairtrade* _______

Madogu *no-fairtrade* _______

Menika *no-fairtrade* ______

*Vielen Dank für diese Angaben!*

[„Dear participant,

this is a little consumer survey for different new names of sorts of chocolate bars. Some of them are fair-trade and some are not fair-trade. Please read each of the names and the additional information and then report spontaneously how much you would be willing to pay for the given chocolate. The chocolate bar refers to a normal bar of 100 grams weight.

Please write down the value in cents from 0 (if you would not be willing to pay anything for it) to 500 (this is, 5 Euros).

Please report the price in cents, for, instance, 155 for €1.55. Just write down the value into the blank space provided beside each of the names.

[…]

Thank you for these reports”]
